# Gold–Thiolate Nanocluster Dynamics and Intercluster Reactions Enabled by a Machine Learned Interatomic Potential

**DOI:** 10.1021/acsnano.4c03094

**Published:** 2024-07-10

**Authors:** Caitlin A. McCandler, Antti Pihlajamäki, Sami Malola, Hannu Häkkinen, Kristin A. Persson

**Affiliations:** † Department of Materials Science and Engineering, University of California Berkeley, Berkeley, California 94720, United States; ‡ Materials Science Division, Lawrence Berkeley National Laboratory, Berkeley, California 94720, United States; § Department of Physics, Nanoscience Center, University of Jyväskylä, FI 40014 Jyväskylä, Finland; ∥ Department of Chemistry, Nanoscience Center, University of Jyväskylä, FI 40014 Jyväskylä, Finland; ⊥ Molecular Foundry, Lawrence Berkeley National Laboratory, Berkeley, California 94720, United States

**Keywords:** Nanocluster, Interatomic Potential, Molecular Dynamics, Isomers, Coalescence, Gold

## Abstract

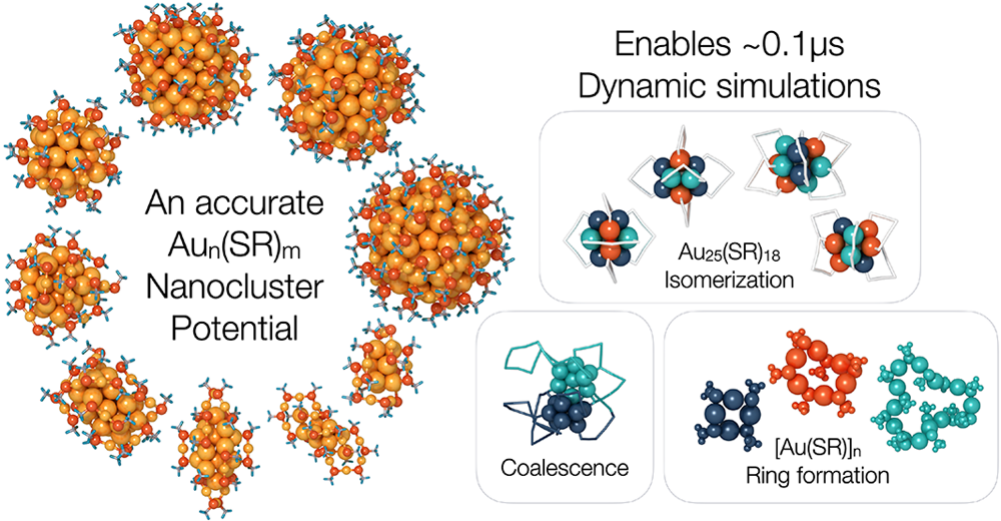

Monolayer protected metal clusters comprise a rich class of molecular systems and are promising candidate materials for a variety of applications. While a growing number of protected nanoclusters have been synthesized and characterized in crystalline forms, their dynamical behavior in solution, including prenucleation cluster formation, is not well understood due to limitations both in characterization and first-principles modeling techniques. Recent advancements in machine-learned interatomic potentials are rapidly enabling the study of complex interactions such as dynamical behavior and reactivity on the nanoscale. Here, we develop an Au–S–C–H atomic cluster expansion (ACE) interatomic potential for efficient and accurate molecular dynamics simulations of thiolate-protected gold nanoclusters (Au_*n*_(SCH_3_)_*m*_). Trained on more than 30,000 density functional theory calculations of gold nanoclusters, the interatomic potential exhibits *ab initio* level accuracy in energies and forces and replicates nanocluster dynamics including thermal vibration and chiral inversion. Long dynamics simulations (up to 0.1 μs time scale) reveal a mechanism explaining the thermal instability of neutral Au_25_(SR)_18_ clusters. Specifically, we observe multiple stages of isomerization of the Au_25_(SR)_18_ cluster, including a chiral isomer. Additionally, we simulate coalescence of two Au_25_(SR)_18_ clusters and observe series of clusters where the formation mechanisms are critically mediated by ligand exchange in the form of [Au–S]_*n*_ rings.

## 1 Introduction

Protected nanoclusters—small atomically precise nanoparticles stabilized by organic ligands—are promising materials for catalysis, optics, and biological sensing.^1−5^ Tailoring their properties necessitates not only establishing structure–property relationships but also guiding synthesis toward narrowly disperse solutions of specific sizes and morphologies. However, while many thiolate-protected nanoclusters have been synthesized and crystallized, the temporal and spatial resolution of the current best characterization techniques limit the ability to image synthesis over time or to observe the dynamic solution-exchange events that contribute to processes such as coalescence. Similarly, *ab initio* molecular dynamics (AIMD) simulations are limited to subnanosecond time periods due to computational cost.

Recent developments in interatomic potential architectures and computational packages show promising results for increased system size and time scale of atomic simulations while maintaining density functional theory (DFT) level accuracy.^6−10^ In this work, the atomic cluster expansion (ACE) interatomic potential formalism was selected due to its speed, accuracy, and ability to extrapolate atomic-level interactions.^11,12^ ACE potentials predict energies and atomic forces from a sum of the atomic interactions present in the structure, including multibody interactions, while also considering the symmetries of atomic interactions to reduce the total number of functions required to represent the system. The overall computational scaling is therefore linear with respect to the system size, and as such, the evaluation speeds rival those of classical potentials while enabling bond breaking and formation, a feat not viable for classical force fields. Furthermore, in comparison to previously fitted interatomic potentials, specifically for gold thiolate clusters,^13−17^ ACE potentials are well positioned to produce accurate dynamical behavior as atomic forces are predicted in addition to total energies. Additionally, each atom is only classified by its element and position, with no bonding enforced between atoms or subclassifications specifying if the atom is a member of the ligand shell or the cluster core. Importantly, exchange between core and ligand gold constituents is possible.

Trained on more than 30,000 DFT calculations of gold nanoclusters, the Au–S–C–H ACE potential successfully reproduces *ab initio* level structure–energy properties of nanoclusters with a variety of atomic packings of the gold core (icosahedral, decahedral, FCC, etc.) and with sizes up to 144 Au atoms. Gas-phase simulations approximate the solvated nanocluster behavior and give insight into nanocluster dynamics that cannot be understood from their crystallized structures. By limiting the cases to neutral thiolated (SCH_3_) clusters in vacuum environments, the potential shows applicability due to its accuracy, force prediction, speed, and facilitation of bond making and breaking. This development allows us to probe long-time-scale dynamical events that are beyond reach of DFT methods. In particular, long finite-temperature simulations of Au_25_(SCH_3_)_18_, up to 0.1 μs time scale, evidence a transformation from the ground state structure into several unique Au_25_(SCH_3_)_18_ structural isomers. Chirality, a property not previously predicted to be possible in the Au_25_(SCH_3_)_18_ system, is observed to be accessible through reversible low-energy transformation pathways. Furthermore, simulations of agglomeration and coalescence of two Au_25_(SCH_3_)_18_ clusters reveal the importance of higher-energy reactive isomers as key intermediates in initiating coalescence. Finally, gold–thiolate rings are found to facilitate size and shape equilibration of the reaction products, as well as atom exchange between clusters.

## 2 Results/Discussion

### 2.1 Validation of the Potential: Structural, Vibrational, and Dynamical Properties

A high degree of accuracy is required to obtain reliable dynamical behavior. We compare a range of properties obtained for known thiolate protected nanoclusters using the ACE potential against *ab initio* predicted energies, forces, vibrational modes, and transition state barriers to benchmark the capabilities of the potential in a range of different aspects.

#### 2.1.1 Structure-Energy Landscape

**Figure 1:**
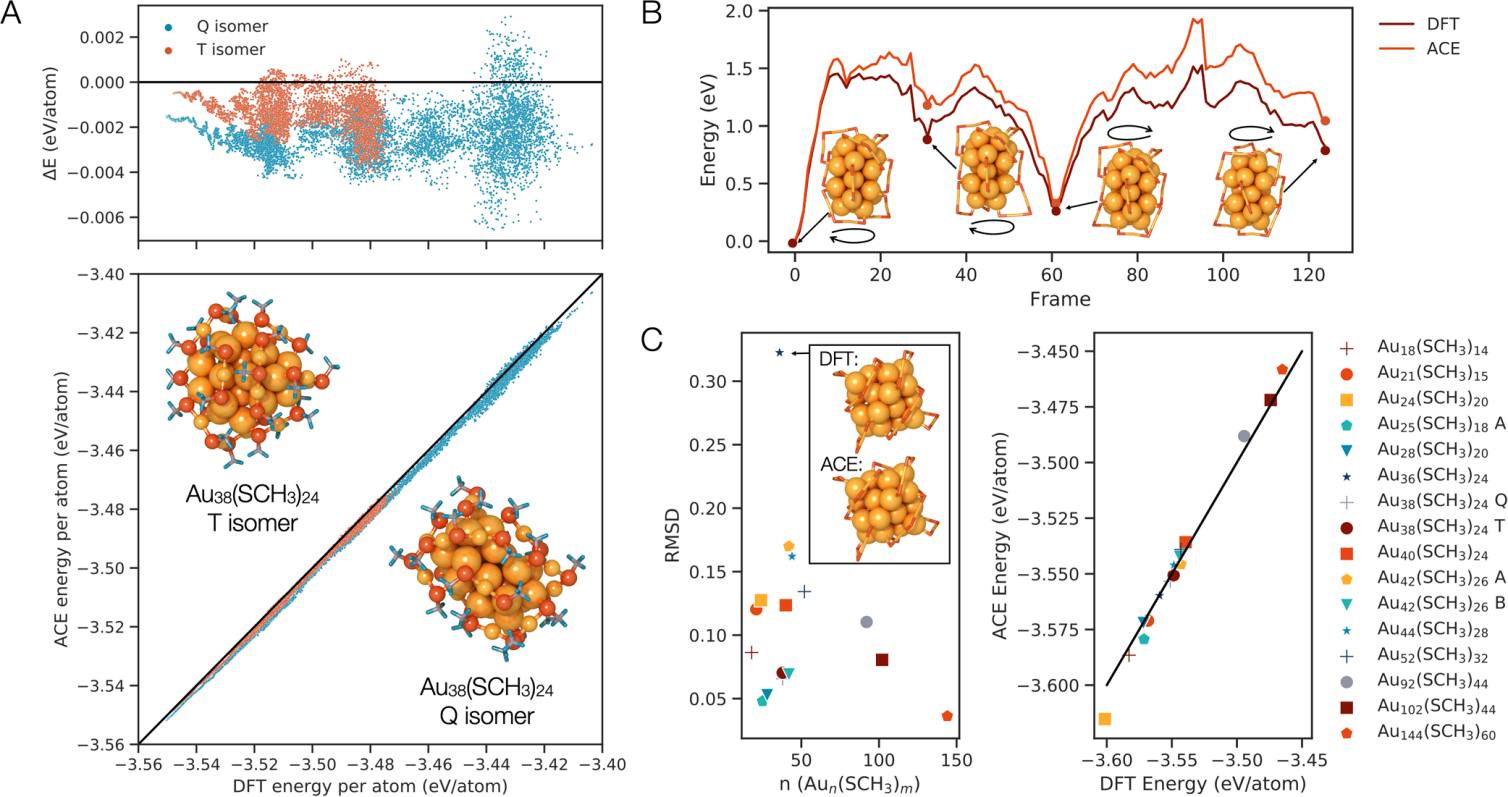
(A) Testing data parity plot, with the initial structures used for the testing simulations (Au_38T_ and Au_38Q_) depicted, as well as the test error, Δ*E*, for the same simulations plotted above. The AIMD test runs are for Au_38T_ up to 500 K (4049 steps) and for Au_38Q_ up to 800 K (7653 total steps). (B) Chiral transition state energies for the twisting of the Au_38Q_ isomer. (C) Crystal structures of known nanoclusters relaxed in the ACE potential have comparable (left) RMSD and (right) predicted energies with respect to those relaxed with DFT.

As described in the Methods section, the training and testing data comprise a collection of molecular dynamics trajectories of Au_38_ isomers obtained from previous studies, as well as clusters with a variety of sizes sampled with the active learning process. In summary, 31,942 structures were used for training, and 11,702 structures were used for testing. The root mean squared error (RMSE) for the testing and training data sets are 2.27 meV/atom and 11.0 meV/atom, respectively (Figure 1A). The testing error is lower than the training error likely due to the fact that the testing data consist of structures generated using AIMD, while the training data include some structures that deviate from equilibrium geometries, hence the standard deviations of testing and training data are 34 meV/atom and 56 meV/atom, respectively (see *Ab Initio* Training Data). A previously fitted extreme minimal learning machine (EMLM) model achieved an RMSE of 16.9 meV/atom for the same test set.^16^ The RMSEs of the forces for the testing and training data sets are 121.1 meV/Å and 87.83 meV/Å, respectively, which correspond to 14% and 22% error in the respective forces.

We further test the ability of the ACE potential to reproduce the DFT geometries and energies of known neutral gold thiolate nanoclusters, sourced from single crystal X-ray diffraction (XRD) coordinates reported in the literature, with their thiol ligands (SR) replaced with methanethiol (SCH_3_; Table 1).^18−35^ Each of these reference clusters was separately relaxed with DFT and with the ACE potential. We note that there will always be slight distortions between the XRD coordinates and those predicted by DFT, especially given that the bulky SR groups are replaced here by SCH_3_. The deviation between the relaxed geometries with the two methods are calculated by their root-mean-square displacements (RMSD) of the Au, S, and C atoms calculated with the Kabsch–Umeyama algorithm implemented in *pymatgen* (Figure 1C).^36^ Thiolate-protected gold nanocluster cores can adopt a variety of atom packing types and most commonly adopt face centered cubic (FCC) or icosahedral geometries. The training structures, Au_38T_ and Au_38Q_, have icosahedral centers, as does Au_25_ and Au_144_ (except for the central gold atom). Many of the structures considered here exhibit FCC cluster cores, including Au_21_, Au_28A_, Au_28B_, Au_36_, Au_40_, Au_42A_, Au_44_, Au_52_, and Au_92_, and even decahedral cores, including Au_102_. The other clusters have structures that are difficult to define by their packing type. Au_36_, which presents an FCC core, showed the least agreement between ACE and DFT, with an RMSD of 0.32, and its DFT predicted structure is shown in contrast to its ACE predicted structure in Figure 1C. The structure differences are difficult to identify by eye, indicating that the deviation even in the worst case is low. Performing the structural optimization with the ACE potential therefore yields similar results to performing the structural optimization with DFT, at a fraction of the computational cost.

**Table 1:** Comparison of Relaxed Structure Energies of Known Neutral Gold Thiolate Nanoclusters from the Literature^18−35^^,^^a^

				cohesive energy prediction error (meV/atom)	
name	ref	formula	methylated formula	DFT optimized	ACE optimized
Au_18_	(18), (35)	Au_18_(SC_6_H_11_)_14_	Au_18_(SCH_3_)_14_	–2.0	–3.7
Au_21_	(19)	Au_21_(S^*t*^Bu)_15_	Au_21_(SCH_3_)_15_	–0.2	–2.7
Au_24_	(20)	Au_24_(SCH_2_Ph^*t*^Bu)20	Au_24_(SCH_3_)_20_	–10.1	–13.8
Au_25A_	(21)	Au_25_(PET)_18_	Au_25_(SCH_3_)_18_	–6.4	–8.0
Au_28_	(22)	Au_28_(SC_6_H_11_)20	Au_28_(SCH_3_)_20_	1.5	–0.2
Au_36_	(23)	Au_36_(SPh)_24_	Au_36_(SCH_3_)_24_	5.5	–0.3
Au_38Q_	(24)	Au_38_(PET)_24_	Au_38_(SCH_3_)_24_	–0.4	–2.1
Au_38T_	(25)	Au_38_(PET)_24_	Au_38_(SCH_3_)_24_	0.1	–2.0
Au_40_	(26)	Au_40_(o-MBT)_24_	Au_40_(SCH_3_)_24_	6.9	3.7
Au_42A_	(27)	Au_42_(TBBT)_26_	Au_42_(SCH_3_)_26_	2.4	–2.6
Au_42B_	(28)	Au_42_(TBBT)_26_	Au_42_(SCH_3_)_26_	3.5	1.5
Au_44_	(29)	Au_44_(TBBT)_28_	Au_44_(SCH_3_)_28_	7.2	2.4
Au_52_	(30)	Au_52_(PET)_32_	Au_52_(SCH_3_)_32_	7.9	4.1
Au_92_	(31)	Au_92_(TBBT)_44_	Au_92_(SCH_3_)_44_	10.1	6.3
Au_102_	(32), (34)	Au_102_(p-MBA)_44_	Au_102_(SCH_3_)_44_	4.6	2.1
Au_144_	(33)	Au_144_(SCH_2_Ph)_60_	Au_144_(SCH_3_)_60_	8.1	6.9

a The thiol ligands in the reported crystal structures of each nanocluster are here replaced with methanethiol (SCH_3_), and their geometries are relaxed with a structural optimization, either using DFT or ACE as the calculator. The ACE energy of the DFT-relaxed structure was compared to the DFT energy of the DFT-relaxed structure (DFT optimized error), and the ACE energy of the ACE-relaxed structure was compared to the DFT energy of the DFT-relaxed structure (ACE optimized error). The training examples mainly consist of simulated data from Au_38T_ and Au_38Q_, and as such, the prediction errors for those clusters are relatively low. The potential can represent nanoclusters with a wide variety of gold core structures and sizes apart from the Au38 clusters, however with slightly higher expected error. PET = phenylethanethiolate, TBBT = 4-*tert*-butylbenzenethiolate, o-MBT = 2-methylbenzenethiolate, p-MBA = *para*-mercaptobenzoic acid.

#### 2.1.2 Transition States

One of the most difficult regions to map in the potential energy surface (PES) of any structure is the transition state, or saddle point, along the trajectory between two metastable structures, or PES local energy minima. As the majority of training data lie within potential energy wells, extrapolations must be made to predict the height of the saddle point separating energy minima. Here, we assess the accuracy of the potential in predicting the energies over a transition pathway between the chiral enantiomers of the Au_38Q_ isomer proposed by Malola and Häkkinen,^37^ with DFT reference energies at each step of the transformation (Figure 1B). The trace of the curve has the same characteristic peaks and valleys, albeit with a positive overall shift in the predicted energies. We hypothesize that the energy scaling issue is likely due to the fact that the potential prioritizes learning the forces as opposed to the energies (see Methods section), which will retain the shape of the curve as compared to the DFT calculated one, with a deviation in the true energy. Overpredicting the transition state energy barriers has the effect of systematically reducing predicted reaction rates and equivalently increasing the simulation temperatures at which reactions would occur as compared to AIMD.

#### 2.1.3 Vibrations

**Figure 2:**
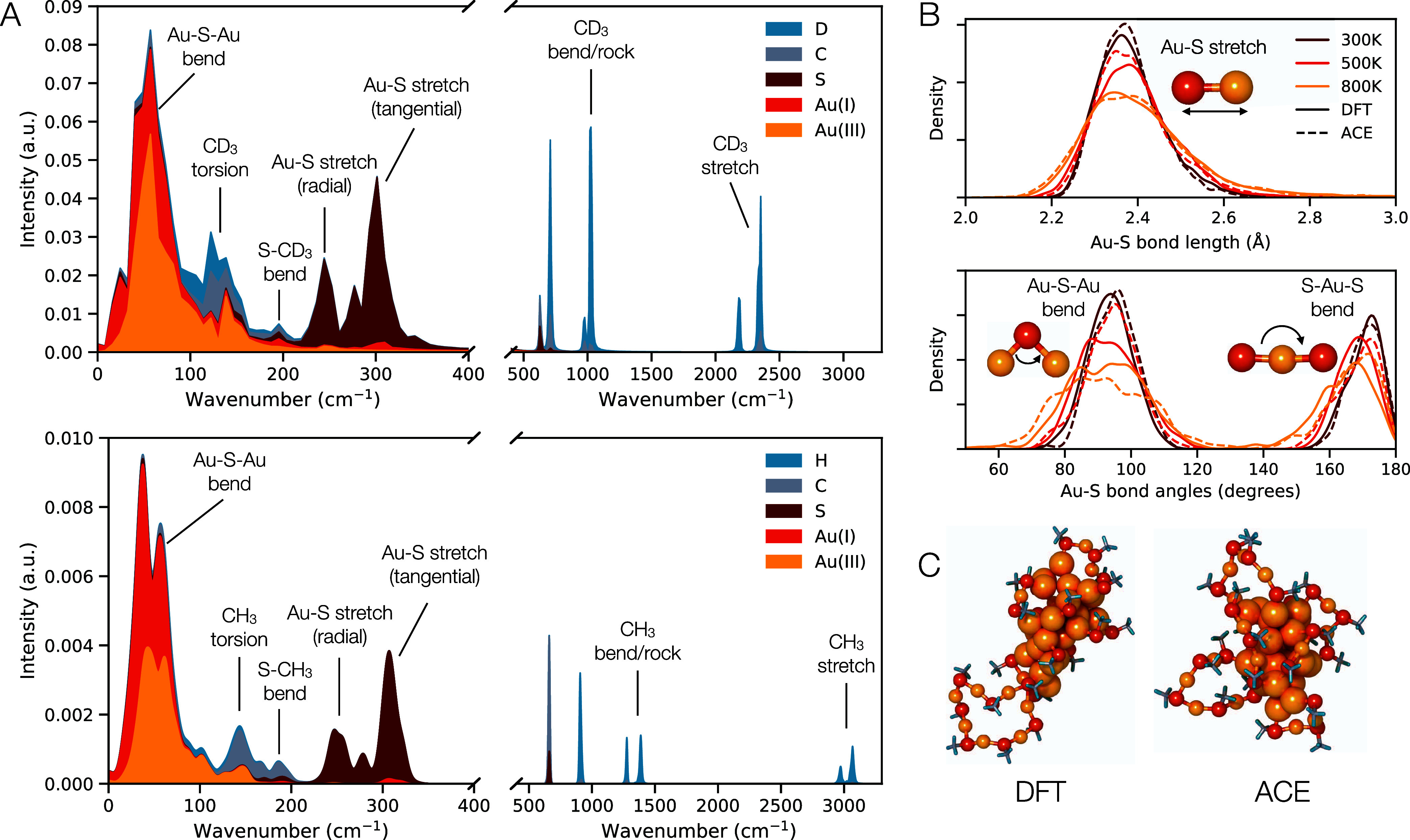
(A) Vibrational density of states of Au_38Q_ at 300 K. Top: AIMD simulated, with hydrogen replaced with deuterium, Langevin dynamics, 1000 steps with 2 fs time steps (0.002 ns simulation) Bottom: ACE simulated, with Langevin dynamics, 90000 steps with 1 fs time steps (0.09 ns simulation). (B) Characteristic bond lengths and angles in the ligand shell, as predicted with DFT and ACE. (C) Snapshots of simulated Au_38Q_ with deuterium at high temperatures. (Left) AIMD simulated using Berendsen dynamics for 0.025 ns, with a final temperature of 1200 K. (Right) ACE simulated using Langevin dynamics for 0.025 ns, with a final temperature of 1200 K.

Vibrational densities of state (VDOS) of the gold–thiolate clusters are recreated with the ACE potential as compared with AIMD. The atom-resolved VDOS calculated for Au_38Q_ with each simulation method are included in Figure 2A. Note that the AIMD simulations use deuterium instead of hydrogen, and as such the vibrational modes that include hydrogen or deuterium are not the same but do match experimentally determined values for similar molecules, like methane and methane-d4. The methane (CH_4_) stretching vibrations exist at 2917 and 3019 cm^–1^, and the bending/rocking modes exist at 1306 and 1534 cm^–1^. The methane-d4 (CD_4_) stretching vibrational modes exist at 2109 and 2259 cm^–1^, and the bending/rocking modes exist at 996 and 1092 cm^–1^.^38^ The heavier deuterium also likely reduces the frequency of the methyl torsional modes at low frequencies. The vibrational density of states in the gold–sulfur core, however, are unaffected by the deuterium and exhibit the same shape as seen in AIMD simulations. The vibrational modes were labeled by comparing these peaks to those observed in computational simulations of other small gold–thiolate nanoclusters.^39^ Visual inspection of the shape of the vibrational peaks for the cluster core confirm the similarity with those calculated for the Au_25_ cluster.^40^ An important feature to encode is the Au–S bonding since the ligand shell behavior depends strongly on the Au participation in the ligand “staple” motif and, indeed, may adopt a charge of Au(I).

Figure 2B compares the distributions of Au–S bond lengths, Au–S–Au bond angles, and S–Au–S angles from AIMD simulations (2 fs/step, Berendsen dynamics) and ACE simulations (1 fs/step, Langevin dynamics) of the Au_38Q_ structure. Although varying time step lengths are implemented to address instabilities when using very long timesteps with the ACE potential, equivalent time frames are sampled to ensure that structures are at comparable stages of deviation from the ideal geometry. The concluding segments of each simulation are utilized to ensure that the temperature reaches equilibrium. In the 300 and 500 K simulations, 1500 steps are skipped, and the final 500 steps are used. In the 800 K simulation, 3000 steps are skipped, and 578 steps used. The average bond length and bond angles are similar regardless of temperature, but the standard deviations of the bond length distributions spread with higher temperature, as expected.

#### 2.1.4 High Temperature Dynamics

We found that high temperature ligand dynamics qualitatively match with what is expected from AIMD simulations, where the Au–S–Au ligand groups form long chains and even rings in the very high temperature DFT. Figure 2C shows a rendering of the final structure of the training AIMD simulation for Au_38Q_,^41^ with variable temperature ramps ending at 1200 K over a simulation of 0.025 ns, alongside a rendering of the same structure heated in a simulation using the ACE potential heated to 1200 K.

### 2.2 Cluster Isomerization and Cluster–Cluster Reactions

#### 2.2.1 Au_25_ Isomerization

In the case of thiolate-protected gold nanoclusters, isomers exhibit different gold cluster cores or simply different arrangements of stabilizing ligands. Isolating and characterizing isomers in experiments is challenging, particularly in the solution phase. In a few cases, crystallization to specific isomer structures has been successful.^24,25,27,28^ Modifying the synthesis conditions, including exchanging the stabilizing ligand chemistry and adding counterions to change the cluster charge state, can induce new isomers to form.^42^ In this work, known clusters will simply be referred to as Au_*n*X_ where *n* denotes the number of gold atoms in the cluster and X optionally encodes the isomer identity. Literature references and experimental ligand chemistry for each cluster are listed in Table 1.

Cao and collaborators have shown that Au_25_(SR)_18_^–1^ clusters (with a water-soluble thiol) can dynamically interconvert between two isomers depending on solvent conditions and surfactant molecules.^43^ The major isomer was successfully characterized in 2008 when its crystal structure (with organo-soluble thiol) was discovered simultaneously by the Murray^44^ and Jin^45^ groups. Another isomer, stabilized by the surfactant and change of solvent, was assigned to a theoretically predicted cluster^46^ and observed in gas phase ion mobility studies.^47^

**Figure 3:**
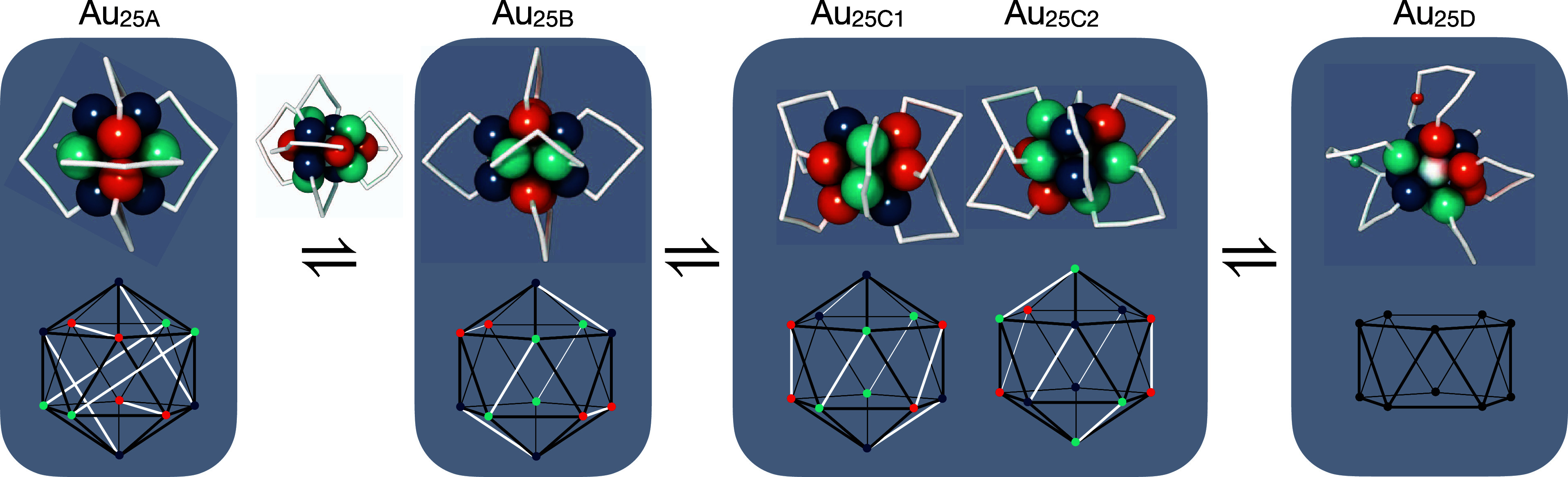
Au_25_ isomerization steps in a finite temperature simulation at 450 K over 42 ns. The ground state structure, Au_25A_, transforms into Au_25B_, which then follows an asymmetric transition into chiral enantiomers Au_25C1_ and Au_25C2_, which further transform into Au_25D_. Atomic renderings maintain the same coloring of each atom over the course of the transformations to help guide the eye, and gold–thiolate units (−SR–Au–SR–Au–SR−) are depicted in white. Schematics under the atomic renderings indicate the bond topology in each isomer, where black lines indicate gold connectivity in the icosahedral cluster core and white lines indicate ligand protecting unit connectivity. The Au_25D_ structure is labile, thus having no fixed conformation of the ligand groups.

Here, we denominate the neutral form of the ground state isomer Au_25A_ and the neutral form of the minor isomer Au_25B_ that has been predicted earlier based on DFT calculations.^46^ Notably, the transformation from Au_25A_ to Au_25B_ is recreated with the interatomic potential. More interestingly, a pair of chiral isomers that have not previously been reported, labeled Au_25C1_ and Au_25C2_, are also observed. These Au_25C_ isomers transform into an additional isomer, Au_25D_, that has only 11 gold atoms in the cluster core and is a possible precursor to multicluster coalescence. A summary of the observed isomerization pathways is presented in Figure 3.

Simulations of Au_25A_ at elevated temperatures (450 K) reveal a temperature-induced transformation between Au_25A_ and Au_25B_ (elevated temperatures are used in order to accelerate the sampling of reactive events). In the Au_25A_ isomer, gold atoms that are linked to one another via protecting SR–Au–SR–Au–SR units are second nearest neighbors within the icosahedron, whereas they become first nearest neighbors in the Au_25B_ isomer. The transformation is initiated by opening the gold cluster core, maintaining two planes of mirror symmetry (Figure S1). While the Au_25B_ isomer exhibits slightly higher energy than the Au_25A_ isomer, it exhibits greater freedom of movement and vibration in the ligand shell, indicating that the Au_25B_ isomer is favored at high temperatures over the ground state Au_25A_ isomer.^46^

Apart from the established isomers Au_25A_ and Au_25B_, we observe a reversible transformation occurring at 450 K, wherein Au_25B_ converts into a previously unreported isomer, Au_25C_. In contrast to Au_25A_ and Au_25B_, the isomer (Au_25C_) identified herein is chiral and further lacks the three mirror symmetry planes. Since both enantiomers easily interconvert between one and the other via the pathway Au_25C1_ ⇌ Au_25B_ ⇌ Au_25C2_ and have nearly degenerate energies, we expect that racemic mixtures would exist (likely also in coexistence with Au_25B_) if their synthesis was not guided by another chiral factor like a chiral ligand or circularly polarized light. Interestingly, Au_25C_ may be an important precursor in the coalescence of multiple Au_25_ clusters, as it transforms into a Au_11_[(SR)_3_Au_2_]_2_[(SR)_6_Au_5_]_2_ (Au_25D_) structure which exhibits an open facet, making it more accessible to incoming reacting species. The transformation of Au_25C_ into Au_25D_ occurs in a two-step process, where in each step two neighboring ligand protecting units (−SR–Au–SR–Au–SR−) together extract one Au atom from the core to construct a longer ligand polymer, [(SR)_6_Au_5_]. Au_25D_ exhibits facile movement of the cluster core, and as such, the decorating ligands quickly swap relative positions without breaking any Au–S bonds. The transformation of Au_25D_ back to Au_25C_ was not observed within the time frame of the simulation (42 ns).

#### 2.2.2 Au_25_ Coalescence and Equilibration

**Figure 4:**
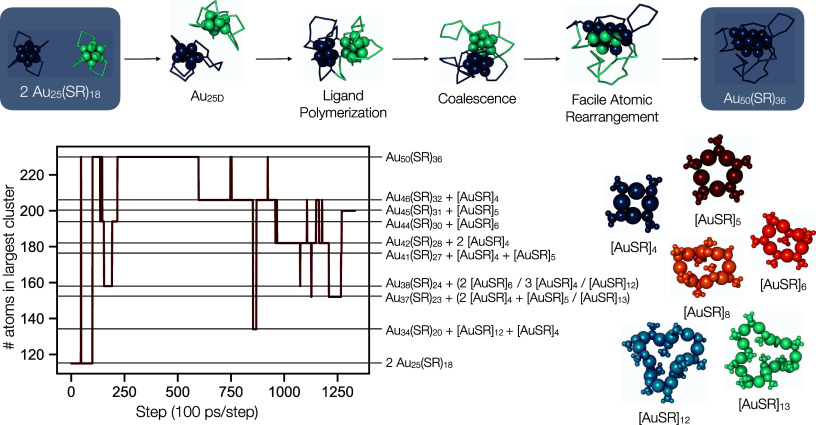
Finite temperature simulations (500 K) of two Au_25B_ clusters show possible mechanisms of coalesence, here proceeding via isomerization into the Au_25D_ isomers, followed by ligand group interaction, and finally the merging of the cluster core. An excess of protecting ligands then leave the cluster as [AuSR]_*n*_ rings with *n* ∈ {4,5,6,8,12,13}. The [AuSR]_*n*_ rings aid in size equilibration and metal exchange between the cluster core and protecting groups. Many sizes of clusters, each with the predictable formula Au_50–*n*_(SR)_36–*n*_ are observed in the 134 ns simulation. The long MD trajectory (134 ns) is sampled by snapshots separated by 100 ps. The composition of the simulated system (main cluster and the fragments) is determined at each snapshot. [AuSR]_8_ and [AuSR]_13_ were observed, but with lifetimes shorter than the 100 ps sampling. Figure S2 shows several snapshots from the simulations.

In order to investigate coalescence, two Au_25B_ clusters were simulated surrounded by vacuum at high temperatures (500 K). The Au_25B_ clusters follow the same individual transformation into Au_25D_, and then interact with each other, initially via the attachment of protecting units (SR-[Au-SR]_*n*_) to the opposite cluster, and then finally via the merging of the cluster cores (Figure 4). The atoms at this temperature show facile rearrangement, with gold atoms that were once part of the core joining the protecting groups, as well as the cluster core mixing easily. The coalesced structure has 50 Au atoms and 36 SCH_3_ ligands, which is more ligands than the core needs to be protected (Au_52_, for example, has 32 SCH_3_ groups, see Table 1). Because of this supersaturation of ligand groups on the cluster core, the polymer-like units tend to pinch together, resulting in [AuSR]_*n*_ ring formation. These rings will coordinate with the cluster core and, from this position, can desorb from the cluster entirely (with desorption defined as the point where all atomic distances are longer than 3.2 Å, 2.8 Å, 2.5 Å, and 2 Å for Au–Au, Au–S, S–C, and C–H bonds respectively). Several [AuSR]_*n*_ ring sizes were observed (*n* = 4, 5, 6, 8, 12, 13), with [AuSR]_4_ being the most commonly formed ring size. Since the ligand rings have a fixed ratio of gold atoms to thiolate groups, a predictable resulting cluster size is formed, Au_50–*n*_(SCH_3_)_36–*n*_. As such, a variety of ligand to gold ratios are achievable: anywhere from Au_50_(SCH_3_)_36_ (*n* = 0) to a bare 14-atom gold cluster (*n* = 36). This equilibration simulation was run for 134 ns, during which time, a variety of clusters were sampled, ranging from Au_50_(SCH_3_)_36_ to Au_34_(SCH_3_)_20_ (0 ≤ *n* ≤ 16). A summary of the sizes sampled over the complete trajectory is included in Figure 4.

Neutral Au_25_(SCH_3_)_18_ in solution has been observed to transform into Au_38_(SCH_3_)_24_ without any coreactants at 65 °C.^48^ In this simulation, the Au_38_(SCH_3_)_24_ cluster size was generated via multiple pathways, including subtracting three [AuSR]_4_, two [AuSR]_6_, or an [AuSR]_12_. Less stable cluster geometries are more likely to have [AuSR]_*n*_ rings add onto the cluster, rearrange, and then leave again. As this simulation duration is relatively short, we hypothesize that, given more time and via this ring-mediated ligand exchange, more stable clusters can be easily achieved, and thus the thermodynamic product will likely form.

The ligand exchange reaction has been observed via labeling different ligand types in solution and observing the ligands exchange over time.^49^ While free monothiol exchange has been postulated to be responsible for the ligand exchange reaction in thiolate-protected nanoclusters,^32,50,51^ our simulations indicate that, in addition to free monothiol exchange, ligand exchange is facilitated via [AuSR]_*n*_ ring exchange. The [AuSR]_*n*_ rings have been observed previously^52−56^ and form here spontaneously upon the merging of two clusters given the relative excess of protecting ligands to the gold core.

## 3 Conclusions

We have developed and validated an interatomic potential in the ACE framework, trained on a large number of DFT calculations, suitable for dynamical studies of ligand-protected gold nanoclusters for time scales that are at least 4 orders of magnitude longer than what is available for traditional AIMD simulations. The potential is accurate over a range of shapes and sizes, can handle reactive chemistry and high temperatures, and may be used to simulate a variety of complex behaviors including crystallization, ligand exchange, agglomeration, and synthesis. Future work may include adding explicit or implicit solvation and partial charges on each atom, for greater accuracy in representing solvated nanoclusters. Similarly, extending the model to chemically varied ligand molecules would give insights into the steric effect on nanocluster behavior. The potential can be upfitted with additional training data and can be considered a successful proof-of-concept to be extended for other ligand-protected metal clusters in the future. To this end, we have opened all training data and essential scripts for the benefit of the community.

Our 0.1 μs time scale simulations yielded rather striking predictions of possible atom-scale mechanisms for high-temperature dynamics of the Au_25_(SR)_18_ cluster and its reactions. Chiral isomerization and subsequent transformation to higher-energy, more reactive isomers is observed, in particular featuring a “polymerized” layer of gold–thiolate units exposing a reactive gold surface that facilitates coalescence. Furthermore, after about 50 ns, the system achieved a “dynamical equilibrium” in which continuous reactions between the coalescent cluster and ring-like [AuSR]_*n*_ fragments took place, leading to the formation of a series of Au_50–*n*_(SR)_36–*n*_ clusters including the well-known Au_38_(SR)_24_ composition.

Our results might offer a key to understand atom-scale mechanisms of Au_*n*_(SR)_*m*_ clusters taking place during energetic processes such as fragmentation reported in electrospray ionization mass spectrometry^57−61^ and ligand removal that is observed during heating of clusters to activate them as catalysts on oxide surfaces.^62,63^

Exchange of ligands and metal atoms between “magic” clusters in the solvent phase has been reported frequently during the past years.^64,65^ Furthermore, NMR studies implied that even metal atoms inside a given “magic” ligand-stabilized silver cluster exchange between all symmetry-unique sites on the time scale (seconds) of the measurement.^66^ Maran’s group reported already in 2018 that “magic” neutral Au_25_(SR)_18_ clusters fuse together in solvent at slightly elevated temperatures to form the Au_38_(SR)_24_ cluster, which implies a greater thermodynamic stability of the latter one.^48^ We have indeed found Au_38_(SR)_24_ as one possible outcome of the Au_25_ fusion reaction in this work, but our current simulation times are too short to establish unambiguously a thermodynamic equilibrium where only the Au_38_(SR)_24_ would dominate. However, all the experimental evidence gathered during the past years indicate that atom-scale dynamics of these types of nanomaterials under ambient conditions in solvents or in the gas phase is still poorly understood, and theoretical developments facilitating reliable long time scale simulations are urgently needed. We hope that our work will enable such investigations on the microsecond timescale and beyond.

## 4 Methods

### 4.1 *Ab Initio* Training Data

The fitted Au–S–C–H potential developed here is highly tuned to the training examples, specifically because gold nanoclusters exhibit molecular-like behavior that is very different from that of the bulk. The training data for this potential were sourced from previous studies on the Au_38_ T and Q isomers^16,41^ that were initially discovered by Tian et al. and Qian et al.^24,25^ A total of 12,413 Au_38Q_ structures and 12,647 Au_38T_ structures were obtained from the Juarez-Mosqueda et al. study and consist of AIMD trajectories with temperatures spanning 500–1200 K, deuderated structures, 2 fs time steps, and Berendsen dynamics.^41^

Additionally, structures were sourced from a study by Pihlajamäki et al., as this study used a different machine learning architecture (EMLM) in order to enable Au–S–C–H simulations via Monte Carlo dynamics.^16^ This set of structures included 5,749 structures that were generated via their Monte Carlo simulations and added to the training data, as well as their testing data set, which consists of 11,702 total structures generated with AIMD simulations of each Au_38_ isomer at various temperatures (300 K, 500 K, 800 K) with deuderated structures, 2 fs time steps, and Langevin dynamics. Here, we use the same testing data set.

In addition to these initial 30,809 training structures, 1,133 structures were systematically generated, calculated with DFT, and added to the training set over the course of the fitting of this potential in a process called active learning (see Active Learning section).

We used the real space DFT code, GPAW,^67,68^ to generate training and testing data. For the sake of consistency, the settings were the same as those used by Juarez-Mosqueda et al.^41^ The chosen exchange-correlation functional was the Perdew–Burke–Ernzerhof functional (PBE),^69^ and grid spacing was set to 0.2 Å. Two versions of GPAW, GPAW 1.3.0 and GPAW 22.8.0, were used to calculate the training data. There are some reference energy differences between the two versions of GPAW, and the benchmarking of these energy differences is included in Supplementary Figure S3.

#### 4.1.1 Active Learning

The AIMD training and testing data sets exhibit strong correlation, meaning that structural snapshots separated by only 2 fs in time contribute minimally to additional information in the model. AIMD data are useful for parametrizing low-energy structures that are very similar to the starting geometry, however high energy structures that are far from equilibrium are unlikely to be captured well using only AIMD training data. Additionally, some interactions never arise over the course of a DFT simulation because they are nonphysical. For example, sulfur atoms are rarely within a few angstroms of other sulfur atoms in the AIMD simulations, since it is not favorable for the ligand groups to come too close together due to repulsive steric interactions. As a result, spurious interactions between sulfur atoms were a major failure mode in general simulations of individual clusters and interacting ligand shells. In order to learn interactions that are not present in the training data, a process called active learning (AL) was employed so that the final model knows how to handle very general, and even nonphysical, interactions.

In AL, the current best version of the potential is used to simulate test geometries, while a metric of the dissimilarity of the structure to the training set is assessed. The ACE potential framework has a metric for this dissimilarity, the extrapolation grade, that is calculated for every atom and measures how different the atomic environment is from those present in the training data.^70^ As simulations are running, structures can be identified as having high extrapolation grade measurements (an extrapolation grade of greater than 1 indicates that the atomic environment extends beyond the bound of atomic environments that are present in the training data), flagged as having especially high maximum extrapolation grades, at which point they can be calculated with DFT and added to the training set to populate these undersampled regions of the PES. A total of 1,133 structures were added to the potential over the course of three rounds of AL. In each round of AL, nonphysical interactions were identified, and these failure modes were addressed and did not occur in subsequent versions of the potential (Supplementary Figure S4).

### 4.2 ACE Potential Architecture

Using the ACE framework, it would be possible to learn any atomic property (e.g., magnetism), but here we use ACE to learn the energies and forces of nanoclusters, so that we may perform MD simulations. Its general nature means that the ACE formalism can be parametrized to have the same form as many common potential architectures, including moment tensor potentials (MTP) and Gaussian approximation potentials (GAP). To construct an ACE potential, we begin by describing atomic environments using spherical harmonics, up to angular momentum *l*_max_ and radial basis functions represented by the *n*_max_ first power-law scaled Chebyshev polynomials.^71^ Atomic interactions are limited to 9 Å, at which point radial components approach zero.

These atomic basis functions are combined into a new set of symmetrically invariant basis functions, *B*. Appropriate coefficients, *c*_*nl*_^(*K*)^, are learned over the course of fitting the potential to best represent the energies of the training examples.
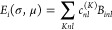
In this equation, *i* represents the atomic index, σ represents the collection of vectors between atom *i* and its neighbors (*r*_1*i*_, *r*_2*i*_,...,*r*_*Ni*_), and μ is the associated list of chemical species of each atom, (μ_1_,μ_2_,...,*μ*_*N*_). *K* represents the multibody order of the interaction, (e.g., an interaction between four atoms would be four-body, *K* = 4). The optimization of the coefficients, *c*_*nl*_^(*K*)^, is performed by minimizing a loss function, Λ, that is given by
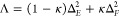
κ determines the relative contribution of the squared deviation in predicted energies, Δ_*E*_^2^, and squared deviation in predicted forces, Δ_*F*_^2^, to the total loss function, Λ. Here, κ is set to 0.9 in order to prioritize learning the forces such that the dynamics of MD simulations would have greater fidelity to the true movements.

Here, we use linear embedding, so the total energy is taken simply as the linear sum of atomic energies. For more detail, we refer the reader to the development of ACE by Drautz.^11^

#### 4.2.1 Body-Order Hierarchical Fitting

With over 1000 basis functions in the target potential structure, finding these optimal coefficients is nontrivial. Thus, the fitting was conducted in a sequential manner, with low body-order fits completed and additional functions added and fit again. During fitting, active learning was also employed in order to improve the training examples and specifically target the regions of the potential energy surface that were the least well represented by the training data.

First, a two-body potential with 125 total basis functions was fitted to a convergence criterion of 1e-8 in the gradient of the loss function. Then, using these learned two-body interaction coefficients as starting values, 950 three-body interactions were added, and this potential was fitted again. Subsequently, the first round of AL was added. Next, 559 Au/S four-body interactions were added, and the potential was fitted again. It is expected that the scaling of accuracy is log–log linear in the number of functions and in the number of training data points;^72^ however, there is a risk of overfitting when using too many basis functions. In total, 1,634 basis functions make up the final potential.

Multibody interactions were indeed necessary for capturing the complex behavior of the gold core and ligand shell. For example, with only three-body interactions, sulfur often bonded to more than two gold atoms, even forming bonds with up to four gold atoms. With four-body interactions, it is less common for sulfur to form more than two gold bonds and may do so as a coordinating bond as opposed to a covalent bond.

The convergence of the final potential was achieved as 0.02 in the gradient of the loss function. The architecture of the final potential is included in Table 2.

**Table 2:** Architecture of Final Potential^a^

elements	max body-order	*n*_max_	*l*_max_
Au–Au	four-body	11/5/5	0/3/3
S–S	three-body	8/3	0/2
C–C	three-body	6/2	0/1
H–H	three-body	4/1	0/1
Au–S	four-body	8/5/4	0/3/3
Au–C	three-body	8/3	0/2
Au–H	three-body	8/3	0/2
S–C	three-body	8/3	0/2
S–H	three-body	8/3	0/2
C–H	three-body	8/3	0/2
All Ternary	three-body	8/3	0/2

a The *n*_max_ and *l*_max_ are reported for each multibody interaction type (i.e. two-body/three-body/four-body).

### 4.3 Molecular Dynamics

All MD simulations were performed with the performant atomic cluster expansion (PACE) implementation in the LAMMPS simulation software (ML-PACE).^12,73^ A Langevin thermostat was used for all MD simulations with the Au–S–C–H ACE potential, using 1 fs timesteps (unless otherwise noted). The evaluation speed for MD simulations of one (two) Au_25_ cluster(s) with LAMMPS is 42 (24) ns/day on a single CPU node (containing two AMD EPYC 7763 Milan CPUs with 64 cores per CPU), which can equivalently be calculated as 0.0021 (0.0036) s/calculation. As compared with the DFT calculations of Au_38_ structures, which were performed on the “Mahti” supercomputer (BullSequana XH2000 system manufactured by Atos) and took approximately 150 s/calculation, ACE offers an approximately 40,000× speedup over DFT. Evaluation speeds may be improved with parallelization across multiple nodes or with the enabled GPU support with the KOKKOS-accelerated implementation of PACE. For more details of the computational cost of PACE with respect to other potentials, we refer the reader to benchmarking completed by Lysogorskiy et al.^12^ All simulation results and nanocluster structures were rendered with Ovito 3.8.3.^74^

Usage note: It is not recommended to use this potential with large time steps, greater than 1 fs/step. In simulations at 500 K with 2 fs time steps, unstable behavior was observed with reasonable starting geometries. High temperature behavior has not been extensively tested, so the recommended maximum temperature would be 500 K, though no instabilities with 1 fs timesteps up to 1200 K have yet been observed in testing.

## Data Availability

The fitted potential, training and testing data sets, and simulation results are all available at https://materialsproject-contribs.s3.amazonaws.com/index.html#ausch_potential/ (DOI: 10.17188/mpcontribs/2356816). The fitted potential (AuSCH_potential.yaml) can be used for simulations in LAMMPS^73^ (https://www.lammps.org) or the Atomic Simulation Environment^75^ (ASE). The coordinates and velocities of the simulation trajectories that are discussed here are also included in the Supporting Information with associated movies showing the full simulations. The potential can be iterated upon and improved with more training data at any time. If readers have an extension they are curious about, they are encouraged to contact the corresponding authors.

## Supporting Information Available

Detailed mechanisms, simulation snapshots, benchmarking and active learning details (PDF)
