# Supplementary material for: Gold–Thiolate Nanocluster Dynamics and Intercluster Reactions Enabled by a Machine Learned Interatomic Potential
Source: ACS Nano. 2024 Jul 10;18(29):19014–23. doi: 10.1021/acsnano.4c03094 (PMC11271183; doi:10.1021/acsnano.4c03094)
Supplement: Supplementary file 1 — nn4c03094_si_001.pdf [file nn4c03094_si_001.pdf]

Supplementary Information: Gold-Thiolate Nanocluster Dynamics and Intercluster Reactions Enabled by A Machine Learned Interatomic Potential

Caitlin A. McCandler[1,2], Antti Pihlajamäki[3], Sami Malola[3], Hannu Häkkinen[3,4,*], Kristin A. Persson[1,5,6,*]

[1]Department of Materials Science and Engineering, University of California Berkeley, CA 94720, USA
[2]Materials Science Division, Lawrence Berkeley National Laboratory, Berkeley, CA 94720, USA
[3]Department of Physics, Nanoscience Center, University of Jyväskylä, FI 40014 Jyväskylä, Finland
[4]Department of Chemistry, Nanoscience Center, University of Jyväskylä, FI 40014 Jyväskylä, Finland
[5]Molecular Foundry, Lawrence Berkeley National Laboratory, Berkeley, CA 94720, USA
[6]Kavli Energy NanoScience Institute, Berkeley, CA 94720, USA
[*]Corresponding authors: Kristin Persson, kapersson@lbl.gov, Hannu Häkkinen, hannu.j.hakkinen@jyu.fi

July 5, 2024

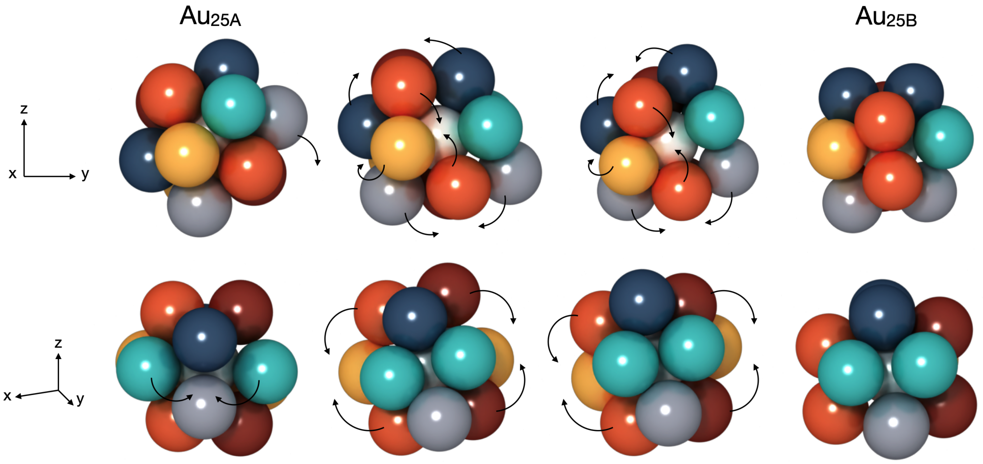

Figure S1: Transition steps between the Au_{25A} and Au_{25B} isomers. Like-colored atoms are connected via SR−Au−SR−Au−SR ligands, which are omitted here for visualization purposes. In the first step of the transformation from the Au_{25A} isomer to the Au_{25B} isomer, two bonded atoms (teal) are pulled together due to the ligand that is attached to each vibrating away from the cluster core, thus forcing another atom (gray) from the cluster core. The transition state induced by this movement has mirror symmetry in the yz plane with an opening in the central atom for subsequent pairs of atoms (red, brown) to come together, with each remaining pair of atoms (yellow, navy) merging in quick succession.

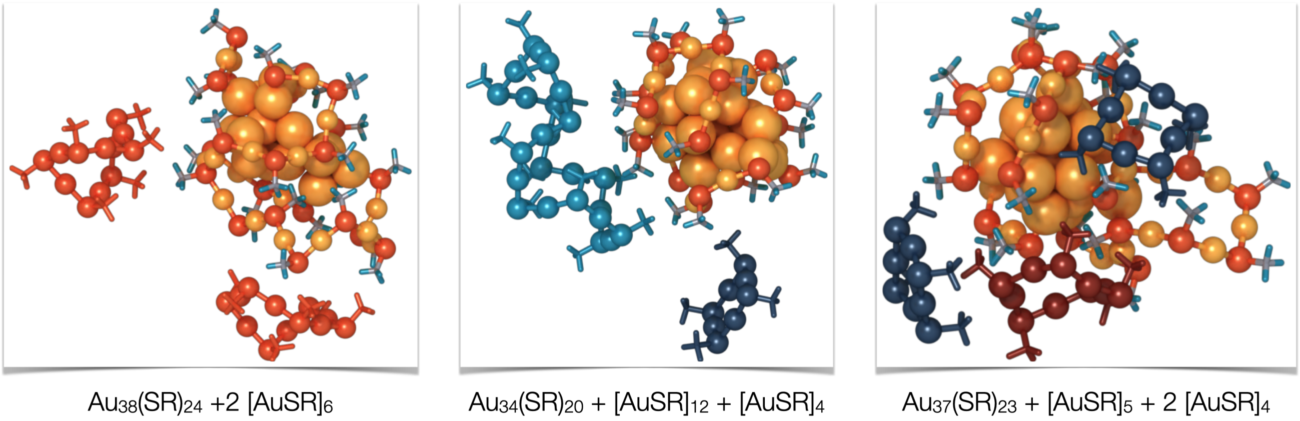

Figure S2: Snapshots of a MD simulation in which two Au_{25B} clusters were heated to 500K, resulting in their coalescence and equilibration into clusters with sizes $Au_{50-n}(SR)_{36-n}$ and $[AuSR]_n$ rings with n $\in$ {4,5,6,8,12,13}

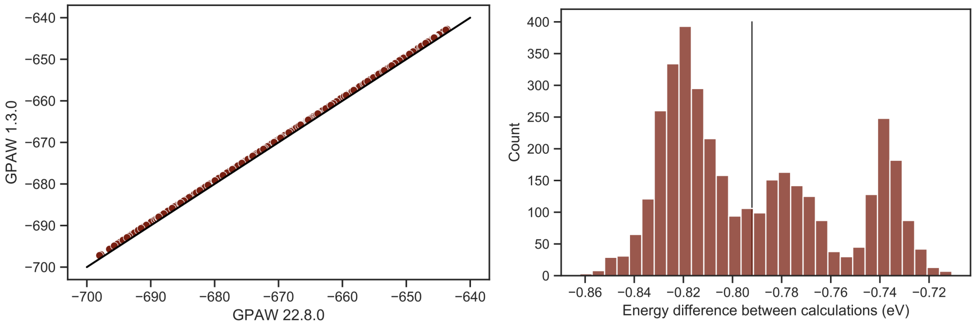

Figure S3: (Left) A systematic energy error was observed due to the versioning of the GPAW simulation code (1.3.0 vs. 22.8.0). In order to reconcile multiple datasets computed with the two versions of the code, benchmarking was performed by recalculating many training structures and quantifying the energy shift (0.79 eV). (Right) Deviations around this shift were observed and may contribute to statistical noise in the training data.

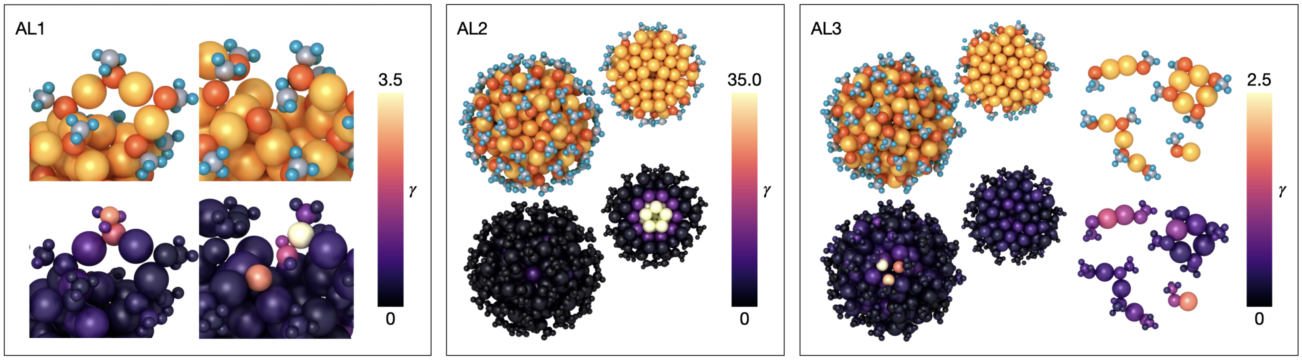

Figure S4: Three rounds of active learning were performed, addressing various non-physical interactions that occurred in testing the fitted potential (γ: extrapolation grade). In the first fitted potential, methyl groups tended to adopt planar configurations between the C and 3 H atoms, with the hydrogens tending to interact non-physically with the S atom. Additionally, methyl groups sometimes jumped from one sulfur to a neighboring thiol ligand. In a subsequent version of the potential, active learning on Au_{144} was performed, since bulk gold was not present in the training data consisting of smaller structures (Au_{38}). The final stage of active learning addressed the tendancy of sulfur groups to coordinate in groups of 3, as well as adding data on small clusters and interacting ligand shells. In each step, the targeted behavior was resolved after active learning.
